# The Effect of the Angiotensin-Converting Enzyme Inhibitor on Bone Health in Castrated Hypertensive Rats Is Mediated via the Kinin-Kallikrein System

**DOI:** 10.1155/2022/9067167

**Published:** 2022-06-14

**Authors:** Na Zhang, Yanan Huo, Chen Yao, Jie Sun, Yafeng Zhang

**Affiliations:** ^1^Medical College of Nanchang University, China; ^2^Department of Endocrinology, Jiangxi Provincial People's Hospital, Nanchang, 330006 Jiangxi, China; ^3^Department of Orthopaedics, The Affiliated Hospital of Nantong University, Nantong City, Jiangsu Province, China

## Abstract

**Background:**

In previous studies, angiotensin-converting enzyme inhibitor (ACEI) use was associated with increased bone loss, while an angiotensin II type I receptor blocker had no effect on bone loss in elder subjects, which suggested that the effect of ACEI on bone loss was not mediated through the classical renin-angiotensin system. In this study, we set to investigate whether the effect of ACEI on bone deterioration was mediated via the kinin-kallikrein system.

**Methods:**

Six-month-old male and female spontaneously hypertensive rats were used. The effect of captopril on blood pressure, serum Ang II, and bradykinin concentration was measured in intact rats. Ovariectomy and orchidectomy were performed to establish an osteoporosis model in female and male rats, respectively. Captopril and the bradykinin receptor blocker icatibant (HOE140) were administered after operation for 12 weeks. Serum Ang II and bradykinin concentration, bone turnover markers, bone mineral density (BMD), and bone microarchitecture were evaluated. Femur samples were subjected to a mechanical test.

**Results:**

Captopril decreased blood pressure and serum Ang II concentration and increased serum bradykinin concentration in intact rats (*P* < 0.05). After castration, captopril decreased serum Ang II concentration (*P* < 0.05); in female rats, icatibant increased serum Ang II concentration (*P* < 0.05). Captopril increased serum bradykinin concentration (*P* < 0.05); in male rats, icatibant decreased serum bradykinin concentration (*P* < 0.05). Captopril increased the rat urine deoxypyridinoline-creatinine ratio (DPD/Cr) and serum osteocalcin concentration (*P* < 0.05). Icatibant decreased urine DPD/Cr in male rats (*P* < 0.05) and increased osteocalcin concentration in female rats (*P* < 0.05). Captopril increased cancellous BMD in castrated hypertensive rats (*P* < 0.05), and icatibant further increased cancellous BMD (*P* < 0.05), which was due to the increased trabecular bone number. In mechanical testing, ACEI increased bone strength (*P* < 0.05), and icatibant further improved it (*P* < 0.05).

**Conclusion:**

ACEI decreased bone deterioration in both male and female hypertensive rats, and the bradykinin receptor blocker further decreased bone deterioration.

## 1. Introduction

Osteoporosis and hypertension are two major chronic diseases in elder subjects [1, 2]. Antihypertensive drugs are widely used to control hypertension in elder subjects. However, many studies have suggested a significant adverse effect of antihypertensive drugs on bone metabolism [3]. Different antihypertensive drugs or even the same type of antihypertensive drugs has been reported to have different effects on bone health. Angiotensin-converting enzyme inhibitor (ACEI) drugs were a typical example [4].

In our previous study, we found that ACEI use was associated with increased bone loss in elder Chinese [5]. Furthermore, we used the multicenter data from America to do the same analysis and found that ACEI use was associated with increased bone loss and the angiotensin II receptor blocker had no effect on bone loss in older male subjects [6]. Since ACEI use was associated with bone deterioration, while the angiotensin II receptor blocker had no effect on bone loss, this suggested that the effect of ACEI on bone loss may not through the classical renin-angiotensin system (RAS).

The kinin-kallikrein system (KKS) is an endogenous multiprotein cascade. This system plays a crucial role in vasodilation, smooth muscle contraction, cardioprotection, vascular permeability, and blood pressure control [7]. ACE also plays a key important role in KKS, which can inactivate bradykinin [8, 9]. It was reported that bradykinin can potentiate cytokine-induced prostaglandin biosynthesis in osteoblasts by enhanced expression of cyclooxygenase 2, resulting in increased RANKL expression [10]. As we know, RANKL can activate osteoclasts and increase bone resorption [11]. Based on these results, we hypothesized that ACEI performs its effect on bone deterioration through KKS by its effect on bradykinin. Furthermore, in a study by Rianon et al., long-term use of ACEI was associated with lower bone loss only in men and not in women [12], which indicated the needs for separate studies in male and female osteoporosis models. So, in this study, we set to investigate whether the effect of ACEI on bone deterioration was performed by KKS using male and female osteoporosis rat models.

## 2. Methods

### 2.1. Animals, Groups, Treatment, and Sampling

Eighty 6-month-old (40 male and 40 female) spontaneously hypertensive rats (purchased from Laboratory Animal Service Center, Nantong University, China) were used in this study. Purified captopril powder was purchased from Zhejiang Huahai Pharmaceutical Co. Ltd. (Linhai, Zhejiang, China). The bradykinin receptor blocker icatibant (HOE140) was purchased from MedChemExpress, USA. The experiment protocol was approved by the Animal Experiment Ethics Committee of the Affiliated Hospital of Nantong University.

Rats were housed in the animal facility at the authors' institute in a controlled environment (temperature: 22°C; 12-hour light and 12-hour dark cycle). All rats were provided *ad libitum* access to standard rat chow and water. 32 rats were used for experiment 1: sixteen male rats were randomly assigned to control and treatment groups (*n* = 8 each): group 1 (control): SHAM with vehicle, and group 2 (ACEI): SHAM with captopril (100 mg/kg/day). Captopril was dissolved in normal saline, and the vehicle and captopril (100 mg/kg/day) were administered daily by intragastric administration for twelve weeks. Body weights of the rats were recorded weekly for adjustment of the dosage of captopril. The same treatments were conducted in female rats.

Twelve weeks after captopril and vehicle administration, urine samples were collected from rats after housing them individually for 24 hours in metabolic cages without providing food and water one day before euthanasia. The rats were anesthetized with combination of ketamine (90 mg/kg) and xylazine (10 mg/kg). Blood sample was collected from the heart for serum isolation. Urine and serum samples were then stored at -80°C before use.

The remaining rats were used for experiment 2: 24 male rats were randomly assigned to the following 3 groups (*n* = 8, each): group 1 (control): castrated plus vehicle; group 2 (ACEI): castrated plus captopril (100 mg/kg/day); and group 3 (ACEI+HOE140): castrated plus captopril (100 mg/kg/day) plus HOE140 (300 *μ*g/kg/day). The same treatments were conducted in female rats. The week of animal death was recorded, and the survival curve was drawn.

SHAM, ovariectomy in female rats, and ORX in male rats were performed at the age of 6 months. Captopril and HOE140 were dissolved in normal saline. The vehicle and captopril were administered daily by intragastric administration from day 4 after operation for twelve weeks. HOE140 was administered daily by abdominal subcutaneous injection from day 4 after operation for twelve weeks. Body weight of the rats was recorded weekly for adjustment of the dosage of captopril and HOE140.

Systolic blood pressure was measured weekly by the tail-cuff method using a noninvasive blood pressure measurement system (ML125 NIBP, AD Instruments, Japan) in conscious rats before operation and during the captopril and HOE140 treatment. The measurement was performed according to the manufacturer's instruction. The protocol was the same as described in our previously published paper [13]. Three repeat measurements were performed at each time point for each rat, and its average value was used for data analysis; the coefficient of variation was 3.33%.

After euthanasia by overdose of ketamine, the left femur was harvested for measurement of BMD and bone microarchitecture was evaluated by micro-CT prior to mechanical testing.

### 2.2. Evaluation of Angiotensin II, Bradykinin, and Bone Turnover Markers

Serum angiotensin II and bradykinin concentrations were measured using a commercially available ELISA kit (Wuhan Fine Biotech Co. Ltd., Wuhan, China), according to the manufacturer's instruction. The coefficient of variation was 4.12%.

Serum osteocalcin (OC) concentration was determined using a commercially available EIA kit (Biomedical Technologies Inc., Stoughton, MA, USA), according to the manufacturer's instruction. Urine deoxypyridinoline (DPD) concentration was determined using a commercially available metra DPD ELISA kit (Quidel, Santa Clara, USA) according to the manufacturer's instruction. The intra- and interassay precision error was 4% and 5%, respectively. Urine creatinine (Cr) concentration was measured using a kinetic Jaffe method on a UniCel DxC600 Clinical System (Beckman Coulter, Fullerton, CA, USA). The calibrators and reagents were prepared according to the manufacturer's instructions. Analytical performance of the method was up to the manufacturer's specifications. Then, urine DPD concentration was corrected with Cr concentration and expressed as a ratio to Cr excretion [14].

### 2.3. Evaluation of BMD and Bone Three-Dimensional (3-D) Microarchitecture

The left proximal femur was scanned with micro-CT (vivaCT-40, Scanco Medical, Brüttisellen, Switzerland) using our established protocol. Briefly, the femoral shaft axis was aligned parallel to the scanning axis. The scanning region included the whole femoral head, femoral neck, proximal femoral shaft, and lesser trochanter with a total scanning length of nearly 10 mm. The scanning was conducted with a resolution of 20 *μ*m per voxel; the segmentation parameters for trabecular bone were fixed at 1.2, 2, and 250 for sigma, support, and threshold, respectively. The parameters for cortical bone were fixed at 10, 9, and 300 for sigma, support, and threshold, respectively. The volume of interest (VOI) of the proximal femoral shaft included the total length of the whole lesser trochanter. The region of interest was drawn semiautomatically in each two-dimensional (2-D) section of the slices of interest.

The microarchitecture and BMD of VOI were automatically evaluated using the built-in program of vivaCT with direct 3-D morphometry. The microarchitectural parameters and BMD were calculated, for trabecular bone including trabecular bone tissue volume fraction (BV/TV), connectivity density, trabecular thickness, trabecular number (Tb.N), trabecular plate separation (Tb.Sp), and trabecular mean density of tissue volume. The coefficient of variation for BMD was 1.57%.

### 2.4. Mechanical Testing

The left femur was used for testing mechanical failure force in a loading configuration designed to stimulate a lateral fall using a custom-made testing device. The distal end of the femur was clamped without ante- or retroversion into a custom-made attachment system. The details of the attachment system are described elsewhere [15]. The femur was fixed against rotation around the diaphyseal axis. The bone was fixed maintaining an angle of 10° between the femoral shaft and the horizontal plane and 15° internal rotation in the femoral neck, as previously demonstrated [16]. A compressive test was performed at a speed of 2 mm/min using a material testing machine with a 2.5 KN loading cell (H25KS Hounsfield Test Equipment Ltd., UK). Failure force was recorded, and energy was calculated using the built-in program.

### 2.5. Statistical Analysis

Firstly, the Shapiro-Wilk test was used to assess the normality of distribution of all variables. All data were expressed as mean ± standard deviation. One-way ANOVA with the LSD post hoc test was used for multigroup comparisons. Statistical power analysis was used to determine the adequacy of sample size. Two-sided *P* values less than 0.05 were considered indicative of statistical significance. SPSS version 11.0 software (SPSS 11.0; SPSS, Chicago) and StataSE software were used for data analysis.

## 3. Results

### 3.1. Animal Information

**Figure 1:**
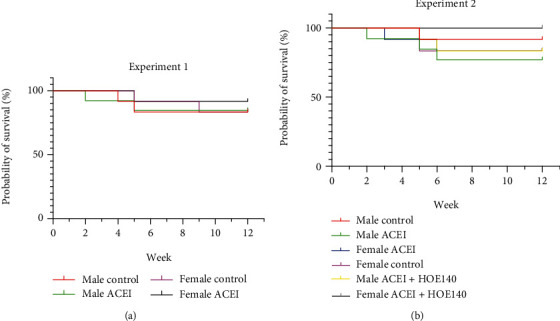
Survival curve of animals of two experiments. There was no difference between different groups in experiments 1 and 2.

Finally, in experiment 1, 6 rats in male group 1, 6 rats in male group 2, 6 rats in female group 1, and 7 rats in female group 2 survived at the completion of 12-week treatment. In experiment 2, 7 rats in male group 1, 5 in male group 2, 6 in male group 3, 6 in female group 1, 5 in female group 2, and 8 in female group 3 survived at the end of 12-week treatment. The result of the survival curve showed that there was no difference between different groups in experiments 1 and 2 (Figure 1).

### 3.2. Effect of ACEI (Captopril) on Blood Pressure, Serum Ang II, and Bradykinin Concentration in Intact Hypertensive Rats

**Figure 2:**
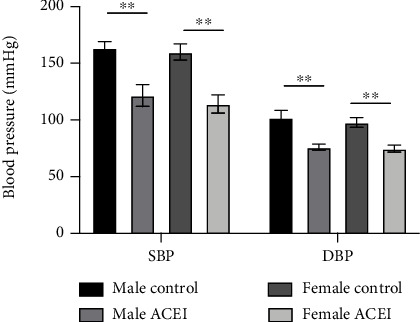
Effect of captopril on blood pressure in intact hypertensive rats. Captopril significantly decreased the systolic and diastolic blood pressure in both male and female intact rats. Data are shown as mean ± SE. ^∗∗^*P* < 0.001. *n* (male control) = 6, *n* (male ACEI) = 6, *n* (female control) = 6, and *n* (female ACEI) = 7.

**Figure 3:**
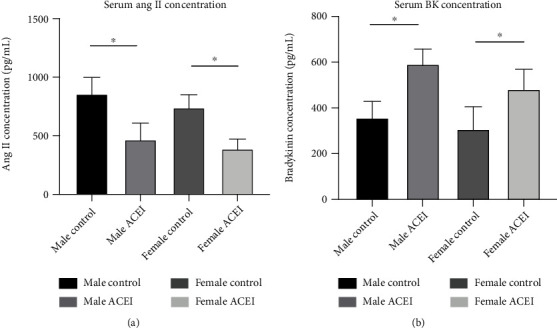
Effect of captopril on serum Ang II (a) and bradykinin (b) concentrations. Captopril decreased Ang II concentration and increased serum bradykinin concentration in intact hypertensive rats. Data are shown as mean ± SE. ^∗^*P* < 0.05, ^∗∗^*P* < 0.001. *n* (male control) = 6, *n* (male ACEI) = 6, *n* (female control) = 6, and *n* (female ACEI) = 7.

In both male and female hypertensive intact rats, captopril significantly decreased the systolic and diastolic blood pressure (male ACEI vs. male control, 121.58 ± 9.67 vs. 163.58 ± 5.45 for systolic blood pressure, 76.08 ± 2.63 vs. 102 ± 6.35 for diastolic blood pressure, *P* < 0.0001; female ACEI vs. female control, 114.07 ± 8.07 vs. 159.91 ± 7.09 for systolic blood pressure, 74.19 ± 3.10 for diastolic blood pressure, *P* < 0.0001) (Figure 2) and serum angiotensin II (Ang II) concentration (male ACEI vs. male control, 468.65 ± 153.19 vs. 857.82 ± 145.56, *P* = 0.0011; female ACEI vs. female control, 388.82 ± 100.60 vs. 741.08 ± 114.53, *P* = 0.0001) and increased serum bradykinin concentration (male ACEI vs. male control, 590.97 ± 65.73 vs. 357.64 ± 71.54, *P* = 0.0002; female ACEI vs. female control, 483.33 ± 85.67 vs. 307.63 ± 97.22, *P* = 0.0053) compared to corresponding controls (Figure 3).

### 3.3. Effect of ACEI (Captopril) and HOE140 on Serum Ang II and Bradykinin Concentration in Castrated Hypertensive Rats

**Figure 4:**
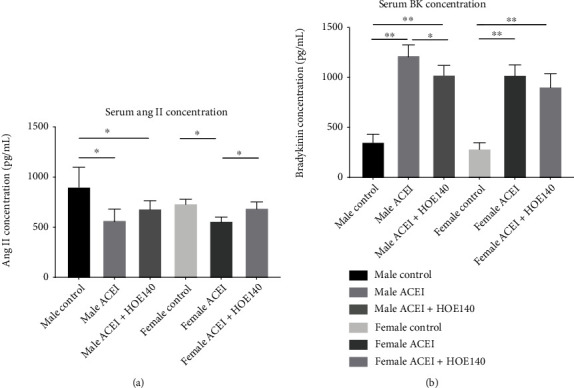
Effect of captopril and HOE140 on serum Ang II (a) and bradykinin (b) concentrations in castrated hypertensive rats. (a) Captopril decreased serum Ang II concentration. In female rats, compared to captopril, HOE140 increased serum Ang II concentration. (b) Captopril increased serum bradykinin concentration. In male rats, compared to captopril, HOE140 decreased serum bradykinin concentration. Data are shown as mean ± SE. ^∗∗^*P* < 0.001. *n* (male control) = 7, *n* (male ACEI) = 5, *n* (male ACEI + HOE140) = 6, *n* (female control) = 6, *n* (female ACEI) = 5, and *n* (female ACEI + HOE140) = 8.

After castration, captopril decreased the serum Ang II concentration. In female rats, compared to captopril, HOE140 caused a significant increase in serum Ang II concentration (female ACEI+HOE140 vs. female ACEI, 703.40 ± 66.68 vs. 563.54 ± 51.42, *P* = 0.0035). In male rats, serum Ang II concentration showed only a decreased trend, but the between-group difference was not statistically significant (male ACEI+HOE140 vs. male ACEI, 686.76 ± 86.10 vs. 571.82 ± 119.52, *P* > 0.05) (Figure 4(a)). Captopril increased the serum bradykinin concentration. In male rats, compared to captopril, HOE140 decreased the serum bradykinin concentration (male ACEI+HOE140 vs. male ACEI, 1032.16 ± 90.08 vs. 1225.71 ± 99.98, *P* = 0.0081). There was only a decreased trend in female rats (male ACEI+HOE140 vs. male ACEI, 888.49 ± 125.23 vs. 1029.88 ± 97.08, *P* > 0.05) (Figure 4(b)).

### 3.4. Effect of ACEI (Captopril) and HOE140 on Bone Turnover Biomarkers in Castrated Hypertensive Rats

**Figure 5:**
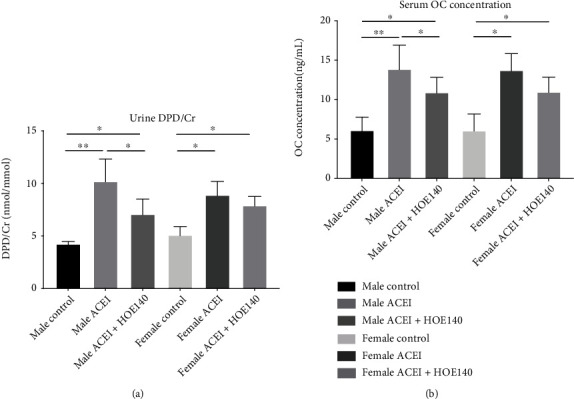
Effect of captopril and HOE140 on bone turnover markers in castrated hypertensive rats. (a) Captopril increased rat urine DPD/Cr concentration. Compared to captopril, HOE140 decreased urine DPD/Cr in male rats. (b) Captopril increased rat serum OC concentration. Compared to captopril, HOE140 decreased osteocalcin concentration in male rats. Data are shown as mean ± SE. ^∗^*P* < 0.05, ^∗∗^*P* < 0.001. *n* (male control) = 7, *n* (male ACEI) = 5, *n* (male ACEI + HOE140) = 6, *n* (female control) = 6, *n* (female ACEI) = 5, and *n* (female ACEI + HOE140) = 8.

In both male and female castrated hypertensive rats, captopril increased rat urine DPD/Cr concentration and serum osteocalcin concentration. Compared to captopril, HOE140 decreased urine DPD/Cr in male rats (male ACEI vs. male control, 10.22 ± 2.10 vs. 4.26 ± 0.23, *P* < 0.0001; male ACEI+HOE140 vs. male ACEI, 7.10 ± 1.42 vs. 10.22 ± 2.10, *P* = 0.0165) and increased osteocalcin concentration in female rats (female ACEI vs. female control, 13.78 ± 2.06 vs. 6.10 ± 2.09, *P* < 0.0001; female ACEI+HOE140 vs. female ACEI, 11.03 ± 1.82 vs. 13.78 ± 2.06, *P* = 0.0077) (Figure 5).

### 3.5. Effect of ACEI (Captopril) and HOE140 on Bone Microstructure and Morphometry in Castrated Hypertensive Rats

**Figure 6:**
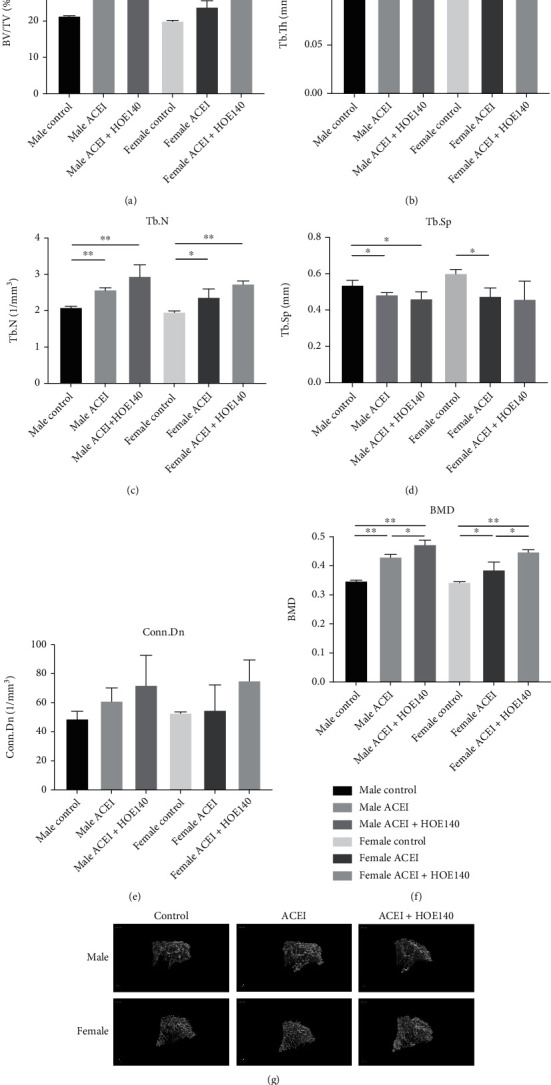
Effect of captopril and HOE140 on cancellous BMD and bone microarchitecture parameters. (a–f) Captopril increased cancellous BMD, BV/TV, and Tb.N and decreased Tb.Sp in both female and male castrated hypertensive rats (*P* < 0.05). HOE140 further increased cancellous BMD and BV/TV. (g) 3-D morphometry of proximal femoral cancellous bone. Data are shown as mean ± SE. ^∗^*P* < 0.05, ^∗∗^*P* < 0.001. *n* = 3.

**Figure 7:**
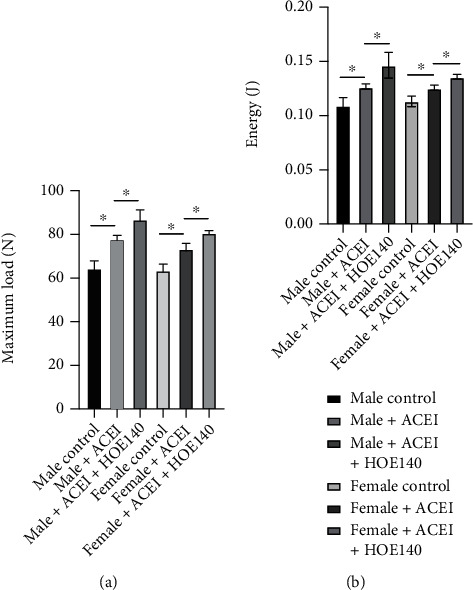
Effect of captopril and HOE140 on bone strength. (a) Captopril increased the maximum load of the proximal femur, and HOE140 further improved it, in both male and female rats. (b) Captopril increased the energy of the proximal femur, and HOE140 further improved it in both male and female rats. Data are shown as mean ± SE. ^∗^*P* < 0.05, ^∗∗^*P* < 0.001. *n* = 3.

Micro-CT data showed that captopril significantly increased cancellous BMD (male ACEI vs. male control, 0.43 ± 0.008 vs. 0.34 ± 0.002, *P* < 0.0001; female ACEI vs. female control, 0.39 ± 0.026 vs. 0.35 ± 0.002, *P* = 0.0475), BV/TV (male ACEI vs. male control, 27.19 ± 0.77 vs. 21.39 ± 0.07, *P* = 0.0002; female ACEI vs. female control, 23.86 ± 1.73 vs. 20.02 ± 0.22, *P* = 0.0186), and Tb.N (male ACEI vs. male control, 2.58 ± 0.05 vs. 2.09 ± 0.03, *P* = 0.0002; female ACEI vs. female control, 2.37 ± 0.23 vs. 1.96 ± 0.03, *P* = 0.0376) and decreased Tb.Sp (male ACEI vs. male control, 0.48 ± 0.01 vs. 0.54 ± 0.03, *P* = 0.0301; female ACEI vs. female control, 0.48 ± 0.05 vs. 0.60 ± 0.02, *P* = 0.0129) in both female and male castrated hypertensive rats (Figure 6). In both female and male rats, HOE140 further increased cancellous BMD (male ACEI+HOE140 vs. male ACEI, 0.47 ± 0.015 vs. 0.43 ± 0.008, *P* = 0.0122; female ACEI+HOE140 vs. female ACEI, 0.45 ± 0.007 vs. 0.39 ± 0.026, *P* = 0.0171) and BV/TV (male ACEI+HOE140 vs. male ACEI, 31.27 ± 1.53 vs. 27.19 ± 0.77, *P* = 0.0145; female ACEI+HOE140 vs. female ACEI, 29.38 ± 1.00 vs. 23.86 ± 1.73, *P* = 0.0088) (Figure 6). According to the microarchitecture data, the increased BMD was attributable to the increased trabecular bone number (Figure 6). Mechanical testing showed that ACEI significantly increased the bone strength in both male and female rats (maximum load, male ACEI vs. male control, 77.77 ± 1.78 vs. 64.43 ± 3.45, *P* = 0.0040; maximum load, female ACEI vs. female control, 73.30 ± 2.61 vs. 63.5 ± 2.86, *P* = 0.0018; compressive test, male ACEI vs. male control, 0.13 ± 0.003 vs. 0.11 ± 0.007, *P* = 0.0227; compressive test, female ACEI vs. female control, 0.13 ± 0.003 vs. 0.11 ± 0.005, *P* = 0.0245). HOE140 further improved the bone strength in both male and female rats (maximum load, male ACEI+HOE140 vs. male ACEI, 86.90 ± 4.37 vs. 77.77 ± 1.78, *P* = 0.0285; maximum load, female ACEI+HOE140 vs. female ACEI, 80.70 ± 1.05 vs. 73.30 ± 2.61, *P* = 0.0103; compressive test, male ACEI+HOE140 vs. male ACEI, 0.15 ± 0.011 vs. 0.13 ± 0.003, *P* = 0.0465; compressive test, female ACEI+HOE140 vs. female ACEI, 0.14 ± 0.003 vs. 0.13 ± 0.003, *P* = 0.0124) (Figure 7).

## 4. Discussion

Short-term use of ACEI has been shown to significantly decrease serum Ang II concentration; however, on long-term use, serum Ang II concentration may return to the baseline levels in patients with left ventricular dysfunction, a phenomenon referred to as “angiotensin escape” [17]. However, our results were not consistent with this theory. In our study, captopril decreased blood pressure and serum Ang II concentration and increased serum bradykinin concentration in intact rats at 12 weeks. This may partially explain why ACEI use in humans was associated with increased bone loss, while in animal studies, ACEI alleviated bone deterioration. Further studies are required to investigate the differential effects of ACEI use on bone loss between human and animals.

The RAS and KKS are endocrine systems that have been reported to be associated with hypertension and osteoporosis. In bone tissues, angiotensin I and II are potent stimulators of osteoclastic activity causing bone resorption [18]. Besides, previous in vitro studies have shown that both bradykinin receptor B1 and bradykinin receptor B2 are expressed on the surface of osteoblasts, while bradykinin can activate osteoblasts and increase the expression of RANKL. In addition, RANKL activates osteoclasts and promotes bone resorption [19]. ACEI can reduce the production of Ang II and the degradation of bradykinin. In our study, captopril increased the serum bradykinin concentration in male rats. Compared to captopril, HOE140 decreased serum bradykinin concentration. According to a previous study, bradykinin increases RANKL expression in osteoblasts, which in turn can activate osteoclasts and result in increased bone loss [20]. However, analysis of our BMD and bone microarchitecture data showed a correlation between increased serum bradykinin concentration and high BMD. This suggests that the actual effect of bradykinin on bone loss may still not be well characterized. Further in vitro study is required to explore the effect of bradykinin on osteoblasts and osteoclasts, and animal study is required to clarify the effect of bradykinin on bone deterioration.

Interestingly, both ACEI and HOE140 increased DPD/Cr and OC concentrations. In male rats, compared to ACEI, HOE140 decreased DPD/Cr concentration and OC concentration, which suggested that HOE140 inhibited osteoclast and osteoblast function in male rats. However, in female rats, there was only a downward trend of DPD/Cr concentration and OC concentration without statistical significance. These results suggest that there may be different underlying mechanisms of bradykinin on bone deterioration between male and female rats. Future study should investigate the underlying mechanisms separately in male and female subjects. These results also suggest that different treatment methods may be required for osteoporosis in male and female patients.

The relationship between RAS and osteoporosis is complex [21]. Our previous cross-sectional study observed a positive association between ACEI use and increased BMD in older people, especially male subjects [22]. This was consistent with experimental studies that showed a detrimental effect of angiotensin II on bone [23]. However, in the same cohort, analysis of longitudinal data revealed an association between ACEI use and increased bone loss [5]. Furthermore, in our analysis of multicenter data from America, ACEI use was found to be associated with increased bone loss while angiotensin II receptor blocker had no effect on bone loss in older male subjects [6]. In our study this time, the effect of ACEI on bone deterioration has been contradicted [12, 24, 25]. A recent study showed that the Ang I-7-ACE2-Mas axis may bypass RAAS and have a positive effect on BMD [26]. Our results and those of previous studies suggest that the effect of ACEI on bone deterioration may be mediated via multiple pathways and need further study.

The strength of this study was the randomized controlled design, focus on the kinin-kallikrein system rather than the classical renin-angiotensin system, and demonstration of differences between male and female rats. The limitations of our study include the small sample size of micro-CT evaluation and the lack of elucidation of the underlying mechanisms of the differences between male and female rats.

In conclusion, in this study, ACEI decreased bone deterioration in both male and female hypertensive rats, and the bradykinin receptor blocker further decreased bone deterioration. This suggested that the effect of ACEI on bone deterioration may be partly mediated via the KKS. The differential effect of HOE140 on bone turnover biomarkers between male and female rats suggested different underlying mechanisms of bradykinin on bone deterioration in male and female rats.

## Data Availability

The data used to support the findings of this study are available from the corresponding author upon request.
